# ECG challenge: ST elevation in critical illness

**DOI:** 10.1093/ehjcr/ytag179

**Published:** 2026-03-12

**Authors:** Michael Gomes

**Affiliations:** Royal Darwin Hospital, 105 Rocklands Drive, Tiwi, NT 0810, Australia

## Clinical vignette

A 26-year-old woman with no prior cardiac history was admitted following an assault resulting in severe traumatic brain injury. Initial imaging demonstrated a depressed left occipital skull fracture with cerebellar intraparenchymal haemorrhage and obstructive hydrocephalus. She underwent urgent operative washout and elevation of the fracture with insertion of an external ventricular drain. Subsequent magnetic resonance imaging showed extensive cerebellar haemorrhage with surrounding oedema, fourth ventricular effacement, and transtentorial cerebellar herniation.

Her intensive care course was complicated by aspiration pneumonia and ventilator-associated pneumonia, requiring prolonged ventilatory support and eventual tracheostomy. She remained under close haemodynamic and neurological monitoring.

**Figure 1:**
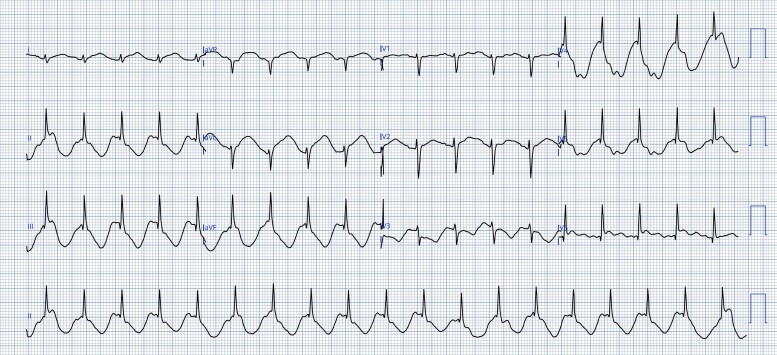
Twelve-lead electrocardiogram recorded at 25 mm/s and 10 mm/mV demonstrating apparent ST-segment elevation with a prominent pre-QRS deflection and dome-shaped morphology in multiple leads.

On Day 3 of admission, persistent sinus tachycardia was noted during routine observation. A 12-lead electrocardiogram (ECG) was obtained (*Figure 1*). The tracing demonstrated marked apparent ST-segment elevation across multiple leads with a striking spike-and-dome morphology and an abnormal deflection preceding the QRS complexes. No prior ECG was available for comparison.

## Question 1

What is the most likely ECG diagnosis?

- Acute anterolateral ST-elevation myocardial infarction (STEMI)
- Hyperkalaemia with pseudoinfarction pattern
- ‘Spiked helmet’ (Pickelhaube) sign
- Acute pericarditis
- Brugada pattern

Correct answer: C

Explanation:

The spiked helmet sign is characterized by apparent ST-segment elevation that begins before QRS onset, producing a spike-and-dome morphology. This timing feature distinguishes it from true STEMI, where ST elevation follows the QRS complex. The pattern is typically transient and most often seen in critically ill patients, usually without myocardial necrosis.^1–3^

## Question 2

Which clinical association is most strongly linked to this ECG pattern?

- Acute coronary plaque rupture
- Critical non-cardiac illness (e.g. intracranial catastrophe and sepsis)
- Congenital channelopathy with fever-induced ST elevation
- Acute viral myocarditis
- Digoxin toxicity

Correct answer: B

Explanation:

The spiked helmet sign has been repeatedly described in association with critical illness, including intracranial haemorrhage, raised intracranial pressure, sepsis, and respiratory failure. It is considered a marker of severe systemic stress rather than primary cardiac pathology.^1,2^

## Question 3

What is the most appropriate next step after identifying this ECG pattern in a stable patient?

- Activate the catheterization laboratory for primary PCI without delay.
- Administer thrombolysis immediately.
- Repeat ECG and correlate clinically; check troponin and perform bedside echocardiography while prioritizing treatment of the underlying illness.
- Start high-dose non-steroidal anti-inflammatory drugs for presumed pericarditis.
- Give intravenous calcium gluconate.

Correct answer: C

Explanation:

In stable patients with atypical ECG changes suggestive of the spiked helmet sign, immediate reperfusion therapy should be avoided unless supported by clinical and biochemical evidence of acute coronary occlusion. Clinical correlation, repeat ECGs, cardiac biomarkers, and echocardiography are recommended while managing the underlying critical illness.^1,3^

**Consent:** Informed written consent was obtained from the patient.

## Data Availability

No new data were generated or analysed in support of this research.
